# Integrative Analysis of Radiation-Induced Senescence-Associated Secretory Phenotype Factors in Kidney Cancer Progression

**DOI:** 10.3390/genes16010085

**Published:** 2025-01-15

**Authors:** Shubhankar Suman

**Affiliations:** Department of Oncology, Lombardi Comprehensive Cancer Center, Georgetown University Medical Center, Washington, DC 20057, USA; ss2286@georgetown.edu; Tel.: +1-202-687-3104

**Keywords:** ionizing radiation, senescence-associated secretory phenotype, renal epithelial cell senescence, renal cell carcinoma, carcinogenesis, ALDH18A1, ASPH

## Abstract

Background: Ionizing radiation (IR) is a well-known inducer of cellular senescence and the senescence-associated secretory phenotype (SASP). SASP factors play dual roles in cancer, either promoting or inhibiting its development. This study investigates IR-induced SASP factors specifically secreted by renal cortical epithelial (RCE) cells and their role in promoting renal cell carcinoma (RCC) progression. Methods: Proteomic data from the SASP Atlas were analyzed to identify IR-induced factors unique to RCE cells, with subsequent evaluations performed at both the gene and protein levels. Thirty-seven proteins were identified as exclusively upregulated and secreted by senescent RCE cells. Gene expression analysis of these RCE-specific SASP factors was conducted using the Gene Expression database of Normal and Tumor tissues (GENT2) and The Cancer Genome Atlas (TCGA). To assess their prognostic relevance in RCC, the corresponding proteins were further analyzed using the Human Protein Atlas (HPA), emphasizing the relationship between SASP factor expression and RCC progression. Results: ALDH18A1 and ASPH emerged as key RCE-specific SASP factors with significant upregulation at both the gene and protein levels (Log2 ratio > 1.15, *p* < 0.05). These proteins are implicated in pro-cancer activities and are strongly associated with poor prognostic outcomes in RCC. Their critical roles in RCC progression underscore their potential as promising therapeutic targets for the prevention and treatment of the disease. Conclusions: This study provides novel insights into the role of IR-induced SASP in renal carcinogenesis, marking the first identification of ALDH18A1 and ASPH as specific secreted proteins associated with tumor progression in RCC. This study suggests that ALDH18A1 and ASPH hold promise as early biomarkers for RCC and as therapeutic targets for disease prevention and treatment.

## 1. Introduction

Ionizing radiation (IR) exposure is well known for causing chronic oxidative stress and DNA damage, activating DNA damage response (DDR) pathways, and inducing cellular senescence [1,2,3,4,5]. A subset of these senescent cells undergoes transcriptional and metabolic reprogramming, acquiring a senescence-associated secretory phenotype (SASP). SASP is characterized by the secretion of a wide array of bioactive molecules, including cytokines, chemokines, growth factors, and proteases, which modulate the tissue microenvironment [6,7,8]. The accumulation of SASP-expressing senescent cells in organs and tissues has emerged as a hallmark of IR-induced accelerated aging and is associated with an increased risk of developing solid cancers [9,10,11]. Recent studies have highlighted the context-dependent role of SASP in cancer initiation and progression. Certain SASP factors exhibit tumor-suppressive effects by promoting the clearance of damaged cells and preventing malignancy, while others contribute to harmful processes such as chronic inflammation, immune evasion, and extracellular matrix (ECM) remodeling, ultimately facilitating tumor progression [12,13,14]. Therefore, distinguishing between the beneficial and harmful components of SASP is crucial for developing targeted interventions that inhibit cancer-promoting SASP factors while preserving their tumor-suppressive effects.

The kidney is a well-recognized radiosensitive organ, where exposure to IR is a significant risk factor for both primary and secondary renal cancer development [15,16,17]. The increased risk of renal cell carcinoma (RCC), the most common type of kidney cancer, has been linked to IR exposure from various radiological diagnostic and therapeutic procedures, particularly in patients undergoing hemodialysis [18,19,20]. Oxidative stress, chronic inflammation, and mutagenesis in cancer-originating cells are commonly implicated in the elevated risk of IR-induced cancers [21,22,23]. While IR exposure causes widespread damage to all cellular components of the kidney, damage to renal cortical epithelial (RCE) cells in the renal cortex is considered a key precursor event for RCC development [24,25,26,27,28]. RCE cells are known for their high proliferation rate and dedifferentiation, undergoing genetic and functional alterations associated with RCC initiation [29,30,31]. These cells are rich in mitochondria and exhibit high metabolic activity, making them particularly susceptible to IR-induced senescence and the acquisition of SASP [32,33,34]. Furthermore, RCE cells reside in a unique tissue microenvironment prone to chronic inflammation, which can further promote tumor growth and progression [35]. Identifying IR-induced, tissue-specific SASP factors, particularly those secreted by senescent RCE cells, could provide novel insights into their role in the pathophysiology of IR-induced renal cancer.

In this study, we conducted an integrative analysis of the SASP secretome of senescent RCE cells, focusing on identifying key SASP factors with potential roles in kidney cancer development. A comprehensive list of IR-induced RCE-specific SASP factors was generated using the SASP Atlas. Furthermore, the expression of SASP-encoding genes in renal cancer was analyzed using the Gene Expression Database of Normal and Tumor Tissues (GENT2) and their prognostic relevance was validated using the Human Proteome Atlas (HPA). By integrating data from the SASP Atlas, GENT2, and HPA, we identified IR-induced, RCE-specific SASP factors that could serve as targets for mitigating the risk of IR-induced renal cancer.

## 2. Materials and Methods

### 2.1. IR-Induced RCE Cell-Specific Soluble SASP Protein Analysis

To identify IR-induced secretory SASP factors specifically originating in RCE cells, we utilized the publicly available SASP Atlas and obtained a comprehensive list of secretory factors associated with IR-induced senescent primary human renal epithelial cells (ATCC #PCS400011) and IMR-90 primary human lung fibroblast (ATCC #CCL-186) cells [34]. As previously described, senescence in both the cell lines was induced by exposure to 10 Gy X-ray radiation, with mock-irradiated cells serving as controls. IR-induced senescent cells were cultured for 10 days and placed in serum- and phenol red-free media for 24 h to collect conditioned media. The conditioned media supernatant, after exosome removal, was used for the soluble SASP (sSASP) characterization via mass spectroscopy-based proteomics analysis [34]. The differentially expressed soluble SASP factor dataset was downloaded from SASP Atlas (http://www.saspatlas.com, accessed on 3 July 2024), and the 10 Gy X-ray-induced senescent cell SASP factors from RCE and IMR-90 cells were compared to identify RCE cell-specific sSASP factors. A cutoff above the Log2 ratio of 1.15 with *p* < 0.05 and no crosstalk with IMR-90 (absent) was designated as an RCE cell-specific SASP factor. Finally, a total of 37 proteins exclusively upregulated and secreted from IR-induced senescent RCE cells were selected for downstream analysis to assess their functional role in renal cancer development.

### 2.2. IR-Induced RCE Cell-Specific SASP Gene Expression in Renal Cancer

The Gene Expression database of Normal and Tumor tissues (GENT2) (http://gent2.appex.kr/gent2, accessed on 25 July 2024) was used to investigate the expression levels of IR-induced RCE cell-specific SASP factors in renal cancer [36]. GENT2 provides large-scale gene expression data, which was utilized to compare the expression levels of SASP factors between normal kidney tissue and RCC samples. A cutoff above the Log2 ratio of 1.15 with *p* < 0.05 was applied to select a list of 14 SASP-encoding genes that were also upregulated in RCC. These genes were subsequently analyzed to evaluate their respective functions at the protein level in renal cancer development.

### 2.3. IR-Induced SASP in Human Renal Cancer

A total of 14 genes with RCE SASP designation and upregulated features at the gene level were evaluated at the protein level using the Human Proteome Atlas (HPA) (https://www.proteinatlas.org, accessed on 20 August 2024) for their prognostic relevance in RCC [37,38]. The prognostic data listed in the HPA database were used to identify factors whose elevated expression is significantly associated with poor prognosis, indicating their potential role in promoting RCC progression. Finally, a cross-referencing analysis was performed to determine tissue-specific SASP signatures and their association with kidney cancer development. In order to validate our findings, RNA-seq data on renal Clear Cell Carcinoma (CCC) and Papillary Cell Carcinoma (PCC) were obtained from The Cancer Genome Atlas (TCGA) database (https://portal.gdc.cancer.gov, accessed on 23 December 2024). The dataset included RNA expression values in transcripts per million (TPM) for 521 CCC patients and 282 PCC patients.

### 2.4. Data Integration and Statistical Analysis

**Figure 1:**
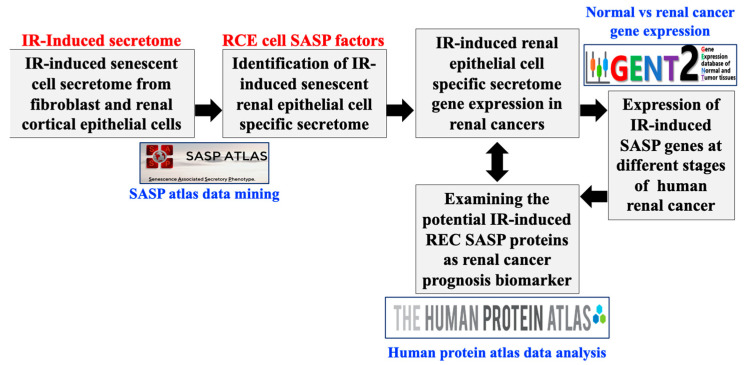
Integrative data-mining approach for the identification of the functional role of the IR-induced senescent renal cortical epithelial (RCE) cell-specific SASP factors in renal cancer development. Our integrative strategy involved SASP Atlas data mining to identify IR-induced RCE cell-specific upregulated SASP factors, followed by comparison of SASP-encoding gene expression patterns between normal and renal cancer tissues and evaluation of IR-induced SASP genes and proteins in parallel to renal cancer prognosis to identify its functional role in renal cancer development. Gene–protein expression and their prognostic value at different stages of renal cancer was conducted using the Human Protein Atlas and GENT2 databases.

To integrate proteomic and transcriptomic data obtained from irradiated RCE cells, the SASP factors with prognostic value identified through HPA analysis were further interrogated using the GENT2 database to establish a correlation between prognostic value and gene expression at different stages of RCC development [36]. The overall study design and data integration approach are illustrated in Figure 1. SASP Atlas database analysis was conducted using Log2 ratio, where relative differences are compared between two specific measurements. The gene expression data accessed from the GENT2 database, represented as Log2 fold change (Log2FC), were converted to a Log2 ratio. For all datasets, statistical analyses were conducted to determine significant differences in gene/protein expression levels between normal kidney tissue and RCC samples. A cutoff above a Log2 ratio of >1.15 with *p* < 0.05 was applied for all statistical analysis. Student’s *t*-test or a Mann–Whitney *U* test was used for two-group comparisons, while ANOVA or a Kruskal–Wallis test was employed for comparisons across multiple cancer stages.

## 3. Results

### 3.1. IR-Induced RCE Cell-Specific Soluble SASP Proteins

**Figure 2:**
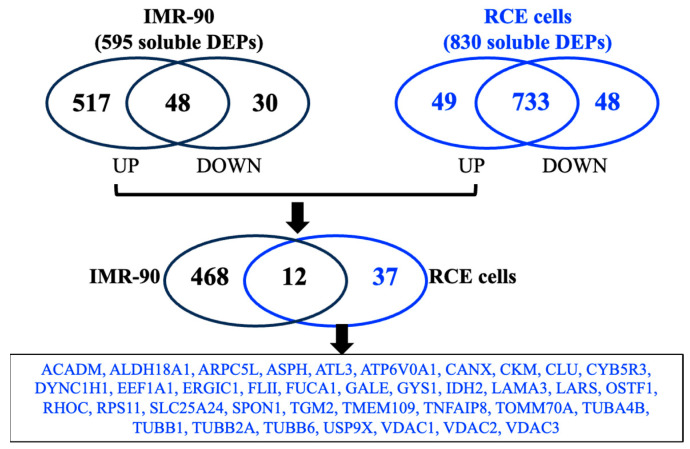
Identification of IR-induced SASP secretome specific to RCE cells (RCE) in SASP Atlas database. Log2 ratios of soluble SASP protein levels from 10 Gy IR-induced senescent cells vs. control cells were obtained for IMR-90 fibroblast (595 differentially expressed proteins from culture media) and RCE cells (830 differentially expressed proteins from culture media). A total of 517 IR-upregulated soluble SASP factors (proteins) from IMR-90 fibroblast cells were compared with 49 IR-upregulated soluble SASP factors (proteins) from RCE cells. Finally, a total of 37 RCE cell-specific IR-induced soluble SASP factors with Log2 ratio >1.15 with *p* < 0.05 were identified and listed.

To identify RCE-specific soluble SASP proteins, we analyzed the IR-induced senescent cell secretome dataset available through the SASP Atlas. The analysis focused on identifying the RCE cell-specific soluble SASP protein levels in the conditioned media of both RCE cells and the reference IMR-90 fibroblast cell line. In the culture media of IMR-90 fibroblast cells, a total of 595 IR-induced differentially expressed proteins (DEPs) were identified, whereas 830 DEPs were detected in RCE cells under similar conditions. From the total secretome, 517 IR-upregulated soluble SASP factors in IMR-90 fibroblasts were compared with 49 IR-upregulated soluble SASP factors identified in RCE cells. After applying a threshold of Log2 ratio > 1.15 and *p* < 0.05, 37 IR-upregulated RCE cell-specific soluble SASP factors were identified (Figure 2). These proteins, unique to RCE cells, represent key components of the RCE-specific IR-induced SASP secretome and are listed in Table 1. This dataset establishes a comprehensive profile of IR-upregulated SASP factors unique to RCE cells, providing insights into tissue-specific responses to IR and senescence.

### 3.2. Status of IR-Upregulated RCE Cell-Specific SASP-Encoding Genes in Renal Cancer

**Figure 3:**
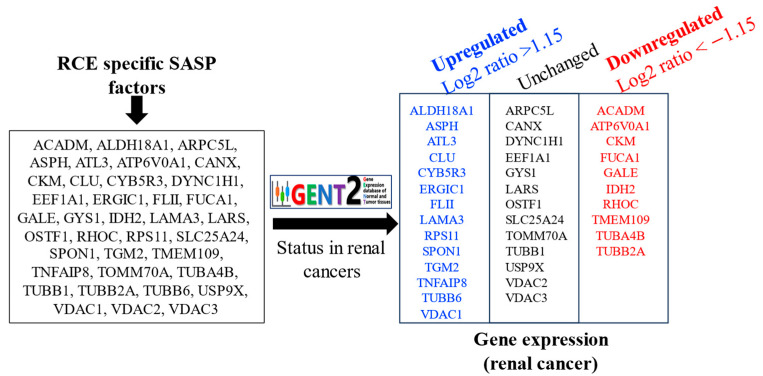
Status of IR-induced RCE cell SASP-factor-encoding genes in renal cancer. A grouping of 14 upregulated (Log2 ratio > 1.15 with *p* < 0.05), 13 unchanged, and 10 downregulated (Log2 ratio < −1.15 with *p* < 0.05) RCE cell-specific SASP genes according to their expression pattens in human renal cancer as reported in the GENT2 database.

The expression patterns of RCE-specific SASP factor-encoding genes were analyzed in the context of human renal cancer using data from the GENT2 database. The 37 RCE-specific IR-upregulated SASP factors identified in this study were categorized based on their reported gene expression trends in renal cancer (Figure 3). A total of 14 SASP genes, including *ALDH18A1*, *ASPH*, *ATL3*, *CLU*, *CYB5R3*, *ERGIC1*, *FLII*, *LAMA3*, *RPS11*, *SPON1*, *TGM2*, *TNFAIP8*, *TUBB6*, and *VDAC1*, were found to be significantly upregulated (Log2 ratio > 1.15, *p* < 0.05) in renal cancer. A total of 13 SASP genes, including *ARPC5L*, *CANX*, *DYNC1H1*, *EEF1A1*, *GYS1*, *LARS*, *OSTF1*, *SLC25A24*, *TOMM70A*, *TUBB1*, *USP9X*, *VDAC2*, and *VDAC3*, showed no significant change in their expression patterns between normal renal tissues and renal cancer. Additionally, 10 SASP-encoding genes, including *ACADM*, *ATP6V0A1*, *CKM*, *FUCA1*, *GALE*, *IDH2*, *RHOC*, *TMEM109*, *TUBA4B*, and *TUBB2A*, were significantly downregulated (Log2 ratio < −1.15, *p* < 0.05) in renal cancer. This classification highlights the dual role of IR-induced SASP factors in renal cancer development, with certain genes potentially contributing to tumorigenesis and others serving as markers of suppressed activity. These findings provide a critical basis for understanding the interaction of tissue-specific SASP factors with cancer pathways.

### 3.3. Role of IR-Upregulated RCE Cell SASP in Renal Cancer Progression

**Figure 4:**
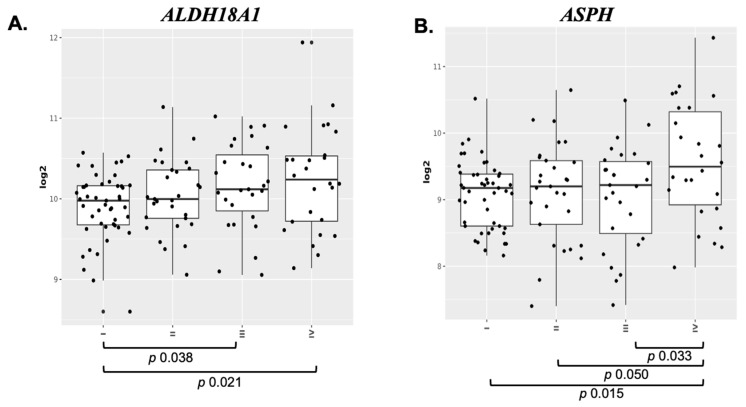
Analysis of IR-altered RCE-cell specific SASP genes in relation to renal cancer stage, analyzed using the GENT2 database. (**A**) *ALDH18A1* expression levels, where statistical analysis revealed significance with *p*-values of 0.038 and 0.021 across different cancer stages. (**B**) *ASPH* expression levels, where statistical analysis revealed significance with *p*-values of 0.033, 0.015, and 0.050 across different stages of renal cancer (I–IV).

**Figure 5:**
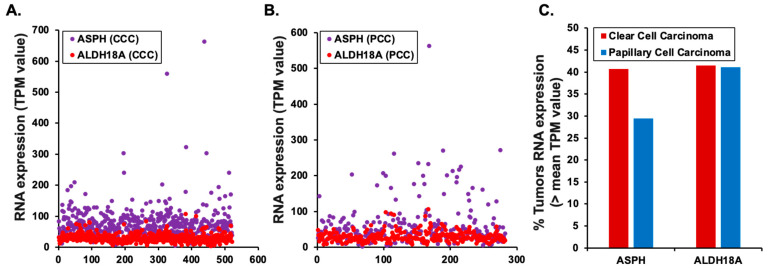
Expression of *ALDH18A1* and *ASPH* in Clear Cell Carcinoma (CCC) and Papillary Cell Carcinoma (PCC) based on RNA-seq data from TCGA. (**A**) RNA expression (TPM) in individual CCC patients. (**B**) RNA expression (TPM) in individual PCC patients. (**C**) Percentage of tumors with RNA expression above the mean TPM value from all tumors of each respective group.

SASP factors exhibit diverse roles, including both tumor suppression and tumor promotion [12]. Therefore, all 14 IR-induced RCE cell-specific SASP factors were examined for their potential roles in renal cancer development through their reported prognostic outcomes, i.e., favorable vs. unfavorable, in the HPA database (Table 2). RCE cell-specific IR-induced SASP factors, such as ALDH18A1 (Log2 ratio 1.43) and ASPH (Log2 ratio 1.20), which are associated with a highly unfavorable prognosis in renal cancers, underscore the tumor-promoting potential of specific SASP factors. In contrast, factors like ATL3 (Log2 ratio 1.40), ERGIC1 (Log2 ratio 1.64), SPON1 (Log2 ratio 1.25), TNFAIP8 (Log2 ratio 1.99), and VDAC1 (Log2 ratio 1.20), associated with a “highly favorable” prognosis, suggest roles in tumor suppression. To correlate and validate the highly expressed SASPs with unfavorable prognostic outcomes, i.e., ALDH18A1 and ASPH, their gene expression patterns were compared at various stages (I-VI) of renal cancer development using the GENT2 dataset (Figure 4). Both *ALDH18A1* and *ASPH* exhibited stage-specific alterations in expression, highlighting their potential relevance to renal cancer progression. Statistical analysis revealed significant changes in *ALDH18A1* expression in stage I vs. stage III and stage IV of renal cancer (Figure 4A). Similarly, *ASPH* expression levels demonstrated significant alterations across stages I–IV of renal cancer (Figure 4B). Further RNA-level analysis of two key RCC types, CCC and PCC, revealed higher expression of the *ALDH18A1* and *ASPH* genes. While *ALDH18A1* was uniformly expressed across CCC and PCC tumors, *ASPH* expression was highly variable in both tumor types (Figure 5A–C). These findings highlight the potential of both *ALDH18A1* and *ASPH* as risk biomarkers and interventional targets for IR-induced renal cancer.

## 4. Discussion

Using SASP Atlas, we identified a subset of 37 IR-upregulated soluble SASP proteins unique to RCE cells. Among these, 14 SASP factor-encoding genes were significantly upregulated in renal cancers. The use of databases, such as GENT2 and HPA, facilitated the further identification of ALDH18A1 and ASPH as critical SASP factors with unfavorable prognostic outcomes, highlighting their tumor-promoting potential. Conversely, SASP factors such as ATL3 and ERGIC1 demonstrated favorable prognostic outcomes, indicating potential tumor-suppressive roles. This dual nature of IR-induced RCE-specific SASP factors underscores their complex contribution to renal cancer pathobiology.

The identification of ALDH18A1 and ASPH as RCE-specific SASP factors suggests a novel link between IR-induced senescence and RCC progression. While ALDH18A1 has been previously implicated in many cancers, including renal cancer [39,40,41], this study uniquely highlights its role as part of the IR-induced SASP, connecting its upregulation to SASP-driven tumorigenesis. Beyond its role in proline metabolism, ALDH18A1 also regulates glutamate metabolism, supporting tumor cell survival under metabolic stress [40]. Additionally, ALDH18A1 has been shown to modulate redox homeostasis, increase resistance to oxidative stress, and promote nucleotide synthesis, all of which are crucial for cancer cell survival and proliferation [39,40]. In kidney and other solid cancer patients, high ALDH18A1 expression often correlates with poor prognosis and therapy resistance [41]. Similarly to ALDH18A1, ASPH is also considered an oncogenic enzyme and is overexpressed in various cancers [42]. ASPH catalyzes the hydroxylation of aspartyl and asparaginyl residues, influencing cell motility, invasion, and metastasis through modulation of ECM interactions and cytoskeletal dynamics [42,43,44]. ASPH also promotes epithelial-to-mesenchymal transition, angiogenesis, and immune evasion, all of which are critical for cancer progression [44]. ASPH is detectable at both the transcriptional and protein levels in human cells, including renal cancer cells, and its elevated expression typically correlates with the development and progression of carcinomas [42,43].

While ALDH18A1 and ASPH involvement in RCC is not novel, their identification as part of the IR-induced SASP and their context-dependent effects provide new insights into their contribution to RCC progression, as demonstrated by their stage-specific expression patterns. These findings align with the established functions of SASP factors in modulating tumor-promoting pathways, such as enhancing inflammation, angiogenesis, and ECM remodeling [45]. Additionally, upregulation of *ALDH18A1* and *ASPH* genes in CCC and PCC suggests their potential as a biomarker and therapeutic target for both key RCC subtypes. Moreover, findings suggesting the tumor-suppressive properties of IR-induced SASP including ATL3 and ERGIC1 also align well with earlier studies [46,47,48]. Specifically, ATL3 expression has been associated with the inhibition of metastatic potential and impaired tumor cell survival [46]. Similarly, ERGIC1 plays a critical role in reducing ER stress, a precursor event linked to the activation of pro-tumorigenic unfolded protein response pathways [47,48]. Furthermore, SPON1 and TNFAIP8 have demonstrated context-dependent effects, with implications in both tumor promotion and inhibition [49,50,51]. These findings extend the current understanding of IR-induced SASP factors that play multifaceted roles in RCC pathobiology.

**Figure 6:**
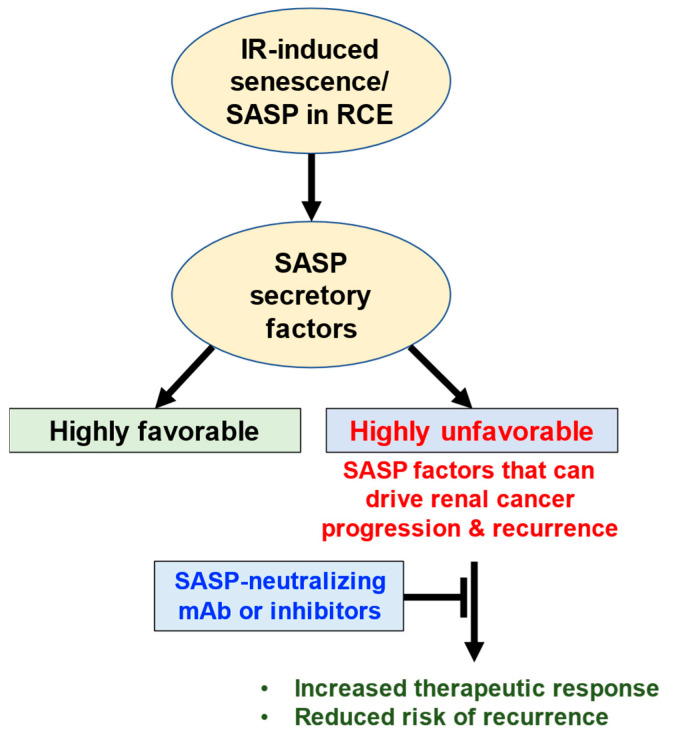
Schematic representation of the impact of IR-induced senescence and senescence-associated secretory phenotype (SASP) in renal cortical epithelial (RCE) cells. IR exposure triggers senescence in RCE, characterized by the secretion of SASP factors. These factors exhibit dual roles: “highly favorable” SASP factors contribute to beneficial outcomes such as tissue repair and immune modulation, while “highly unfavorable” SASP factors promote renal cancer progression and recurrence. Neutralization/inhibition of unfavorable SASP factors using monoclonal antibodies (mAbs) or specific inhibitors could potentially enhance therapeutic efficacy and reduce the risk of renal cancer recurrence.

The integration of IR-induced SASP data highlights the importance of tissue-specific and context-dependent roles of IR-induced SASP factors. As datasets on IR-induced SASP and RCC outcomes expand, future studies can extend and validate these findings. Nevertheless, the current findings align with emerging evidence from other cancers and highlight the dual role of IR-induced SASP factors, specifically their contribution to IR-induced renal carcinogenesis. This study also underscores the importance of distinguishing between favorable and unfavorable IR-induced SASP factors. Highly expressed unfavorable SASP factors, such as ALDH18A1 and ASPH, were identified as key drivers of renal cancer. These molecular insights parallel clinical prognostic factors observed in RCC. For instance, while distant metastasis remains a significant risk factor for postoperative mortality, local tumor spread emerges as a more critical determinant of prognosis [52]. This suggests that molecular drivers like ALDH18A1 and ASPH may contribute to the aggressive local spread and metastatic potential observed in RCC. By linking molecular findings with prognostic implications, this study highlights the need for studies aimed at therapeutic interventions targeting unfavorable SASP factors (Figure 6). Senotherapeutic drugs, which target the detrimental effects of senescence while preserving beneficial functions, could be useful in reducing IR-induced cancer development [53]. Specifically, selective targeting of high-risk SASP factors can be achieved using monoclonal antibodies or small-molecule inhibitors to neutralize their tumor-promoting effects and mitigate the pro-oncogenic environment created by senescent cells [53,54]. The integration of senotherapeutic strategies with existing cancer treatments could further enhance their efficacy [54]. This integrative analysis provides a framework for future investigations into SASP-targeted therapies as adjunctive treatments to mitigate IR-induced renal carcinogenesis.

## 5. Conclusions

This study elucidates the dual role of IR-induced SASP factors in RCE cells, highlighting their complex contributions to tissue homeostasis and tumor progression. Protective SASP factors, such as ATL3 and ERGIC1, play critical roles in maintaining cellular integrity, while detrimental factors, including ALDH18A1 and ASPH, drive a pro-inflammatory and pro-oncogenic microenvironment that can promote renal carcinogenesis. This study offers a novel perspective by identifying ALDH18A1 and ASPH as integral components of the IR-induced SASP, establishing a previously unrecognized link between radiation-induced senescence and renal cancer progression. This dual nature of SASP factors underscores their context-dependent effects, distinguishing between tumor-promoting and tumor-suppressive roles. Importantly, the identification of ALDH18A1 and ASPH as potential biomarkers and therapeutic targets provides a compelling rationale for exploring their clinical relevance. Targeted inhibition of these tumor-promoting SASP factors using monoclonal antibodies or small-molecule inhibitors represents a promising strategy to mitigate renal cancer progression. Future studies should focus on validating these findings in clinical settings and further dissecting the mechanistic pathways underpinning the dual roles of SASP factors. This work lays the foundation for the development of innovative therapeutic interventions aimed at modulating the SASP to improve outcomes in RCC patients.

## Figures and Tables

**Table 1:** Alphabetical list of 37 renal cortical cell-specific IR-induced soluble SASP factors. A cutoff of Log2 ratio (>1.15 with *p* < 0.05) was applied to select this list of IR-induced upregulated SASP factors.

S.No.	RCE Cell-Specific SASP Protein	Log2 Ratio
1	Acyl-CoA dehydrogenase medium chain (ACADM)	1.47
2	Aldehyde dehydrogenase 18 family member A1 (ALDH18A1)	2.91
3	Actin related protein 2/3 complex subunit 5 like (ARPC5L)	1.62
4	Aspartate β-hydroxylase (ASPH)	1.23
5	Atlastin GTPase 3 (ATL3)	1.83
6	ATPase H+ transporting V0 subunit a1 (ATP6V0A1)	1.58
7	Calnexin (CANX)	1.27
8	Creatine kinase, M-type (CKM)	1.43
9	Clusterin (CLU)	1.57
10	Cytochrome b5 reductase 3 (CYB5R3)	1.23
11	Dynein cytoplasmic 1 heavy chain 1 (DYNC1H1)	1.20
12	Eukaryotic translation elongation factor 1 α 1 (EEF1A1)	1.31
13	Endoplasmic reticulum–Golgi intermediate compartment 1 (ERGIC1)	2.67
14	FLII actin remodeling protein (FLII)	2.08
15	α-L-fucosidase 1 (FUCA1)	1.19
16	UDP-galactose-4-epimerase (GALE)	1.27
17	Glycogen synthase 1 (GYS1)	1.21
18	Isocitrate dehydrogenase (NADP(+)) 2 (IDH2)	1.86
19	Laminin subunit α 3 (LAMA3)	1.15
20	Leucyl-tRNA synthetase 1 (LARS)	1.51
21	Osteoclast stimulating factor 1 (OSTF1)	1.70
22	Ras homolog family member C (RHOC)	1.57
23	Ribosomal protein S11 (RPS11)	1.24
24	Solute carrier family 25 member 24 (SLC25A24)	1.20
25	Spondin 1 (SPON1)	1.72
26	Transglutaminase 2 (TGM2)	1.38
27	Transmembrane protein 109 (TMEM109)	1.20
28	TNF α-induced protein 8 (TNFAIP8)	1.45
29	Translocase of outer mitochondrial membrane 70 (TOMM70A)	2.08
30	Tubulin α 4b (TUBA4B)	1.22
31	Tubulin β 1 class VI (TUBB1)	1.37
32	Tubulin β 2A class IIa (TUBB2A)	1.32
33	Tubulin β 6 class V (TUBB6)	1.24
34	Ubiquitin-specific peptidase 9 X-linked (USP9X)	1.26
35	Voltage-dependent anion channel 1 (VDAC1)	2.01
36	Voltage-dependent anion channel 2 (VDAC2)	1.55
37	Voltage-dependent anion channel 3 (VDAC3)	2.20

**Table 2:** Alphabetical list of 14 IR-upregulated SASP factor genes in renal cancers. A cutoff of Log2 ratio (>1.15 with *p* < 0.05) was applied to select this list of IR-induced upregulated SASP factors. As reported in Human Proteome Atlas.

S.No.	Gene	Log2 Ratio	Renal Cancer Prognosis
01	Aldehyde dehydrogenase 18 family member A1 (*ALDH18A1*)	1.43	highly unfavorable
02	Aspartate β-hydroxylase (*ASPH*)	1.20	highly unfavorable
03	Atlastin GTPase 3 (*ATL3*)	1.40	highly favorable
04	Clusterin (*CLU*)	1.39	--
05	Cytochrome b5 reductase 3 (*CYB5R3*)	1.16	--
06	Endoplasmic reticulum–Golgi intermediate compartment 1 (*ERGIC1*)	1.64	highly favorable
07	FLII actin remodeling protein (*FLII*)	1.15	--
08	Laminin subunit α 3 (*LAMA3*)	1.47	--
09	Ribosomal protein S11 (*RPS11*)	1.21	--
10	Spondin 1 (*SPON1*)	1.25	highly favorable
11	Transglutaminase 2 (*TGM2*)	1.37	--
12	TNF α-induced protein 8 (*TNFAIP8*)	1.99	highly favorable
13	Tubulin β 6 class V (TUBB6)	1.84	--
14	Voltage-dependent anion channel 1 (*VDAC1*)	1.20	highly favorable

## Data Availability

We have included all the data in this manuscript or have provided links for publicly available databases where datasets are available for public use.
